# Silica–Acrylic Nanocomposite Coatings for Durable and Hydrophobic Wood

**DOI:** 10.3390/ma19112339

**Published:** 2026-06-01

**Authors:** Andromachi Mitani, Paschalina Terzopoulou, Vasiliki Kamperidou

**Affiliations:** 1 Laboratory of Wood Science and Technology, Department of Forestry, Wood Science and Design, University of Thessaly, 431 00 Karditsa, Greece; amitani@uth.gr (A.M.); zterzopoulou@uth.gr (P.T.); 2 Laboratory of Forest Technology, Department of Harvesting and Technology of Forest Products, Forestry and Natural Environment Faculty, Aristotle University of Thessaloniki, 541 24 Thessaloniki, Greece

**Keywords:** color, conservation, cultural heritage, hydrophobicity, surface modification, silica nanoparticles, roughness

## Abstract

**Highlights:**

- SiO_2_ nanoparticles in acrylic coating enhance wood hydrophobicity and stability.
- Treated *Ailanthus* wood highly resisted weathering and photodegradation.
- Modified wood exhibited significantly lower moisture uptake, improving stability.
- High color stability during aging was achieved with 3–4% nanoparticles present.
- Promising conservation strategy for historical and modern wooden structures.

**Abstract:**

Wood strength, renewability and appearance make it one of the most preferred and widely used natural materials in structural and cultural applications. The gradual degradation of wood from abiotic and biotic factors has an adverse impact on its structural durability and service life. This study investigates the effect of surface treatment of wood of the invasive tree species of tree-of-heaven, through short-term immersion in an acrylic polymer (Paraloid-B72) containing silica dioxide (SiO_2_) nanoparticles at low concentrations (0–4% *w*/*v*) to impart hydrophobic behavior and weathering resistance. FTIR analysis confirmed the successful incorporation of the acrylic polymer and silica nanoparticles within the wood structure without altering the chemical integrity of the substrate. For both treated and untreated wood specimens, the physical properties (density, equilibrium moisture content, surface roughness, color-parameters), hygroscopic properties (swelling/absorption, contact angle) and weathering resistance tests were conducted using xenon-arc combined with wetting–drying cycles. The findings revealed that treated wood has significantly improved hydrophobic performance and dimensional stability, reducing moisture uptake. Treatment significantly increased the samples’ resistance to artificial weathering, with the effectiveness dependent on nanoparticle concentration. Although moderate surface color changes were observed in treated samples (compared to untreated ones), during their exposure to weathering, reduced lightness and slight increases in red and yellow chromatic coordinates were observed, with treated specimens exhibiting higher color stability during aging. Nevertheless, surface roughness increased significantly by the treatment, slightly restricting the method when a highly smooth surface touch is required. The proposed modification method appears promising to prolong the wooden structures’ service-life, meanwhile inspiring modern strategies for conserving historical timber structures that cannot be moved and should be protected by applying less invasive protective methods.

## 1. Introduction

Wood is becoming an increasingly popular sustainable building and engineering material, and research into enhancing its functionality and long-term durability is intensifying. Among the various benefits of wood, its easy processing, good mechanical qualities, and renewability are included. However, the hydroxyl groups in hemicelluloses and amorphous parts of cellulose readily react with moisture, causing hydrolysis, and the subsequent impact of swelling, discoloration, mold growth and decay [1,2]. Wood hygroscopicity is associated with several drawbacks, since variations in relative humidity lead to repeated moisture absorption and desorption, causing internal stresses within the cellular structure, dimensional changes, and surface defects like deformations, cracks and micro fissures [3,4]. All these changes facilitate the degradation of wood structure by bacteria, fungi, and insects [5,6]. Therefore, although wood is gaining attention as a renewable structural material, it remains limited by moisture-induced swelling and shrinking (dimensional changes), surface checking, lignin photodegradation, discoloration, and erosion during weathering [7].

Several factors should be taken into consideration when choosing a protection or modification strategy, including wood species, anatomical structure [8], chemical composition [8], future end-use structural applications [9], exposure environment [10,11], and compatibility with adhesive, fasteners for specific treatments [10,12].

Surface treatment techniques have been more popular in recent decades as simple and efficient ways to increase wood’s resilience to exposure to environmental conditions. Among these methods, acrylic polymers have been widely used in contemporary wood preservation and cultural heritage artifact conservation as a consolidant due to their easy application and wood stabilization potential. Among the most utilized ones, Paraloid B72 is chosen for its reversibility, chemical stability over time, and optical clarity. When applied by immersion or impregnation, this copolymer’s ability to dissolve in a variety of organic solvents (including acetone, ethanol, and toluene) allows for viscosity control through controllable evaporation rates. However, plenty of variables influence its protective performance and the treatment’s efficacy, including temperature, exposure duration, pressure conditions, substrate permeability, adequate penetration depth, and uniform dispersion inside the timber structure.

Previous studies have revealed that Paraloid-based formulations can enter pores and voids and penetrate past the formation of superficial films, but when used alone, they do not offer a strong defense against biological agents, highlighting the urgent need for functional additives to increase durability. New opportunities for wood protection systems have been made possible through nanotechnology. Nanoparticles provide enormous amounts of surface area, high dispersion, chemical stability, and a high affinity for lignocellulosic materials. Because of their tiny size, they can penetrate wood tissues more deeply while preserving low-viscosity compositions [13]. Numerous inorganic nanoparticles, such as ZnO, TiO_2_, SiO_2_, CeO_2_, and FeO_3_, have been investigated either alone or in combination with polymer matrices [14]. For example, the potential of zinc oxide nanoparticles to improve surface hardness and biological protection, as well as to increase resistance to mold, decay, UV radiation and moisture penetration, has been investigated, while other metal-based systems have demonstrated efficacy against insects and fungi [15,16,17] and an increase in biological durability [18,19]. However, ZnO nanoparticles have been widely recognized as highly toxic to aquatic organisms and have been reported to show very high ecotoxicological concern in aquatic systems [13]. SiO_2_ nanoparticles present lower ecotoxicological concern relative to ZnO, as well as optical transparency, and compatibility with polymer matrices [14]. Silica-reinforced polymer composites of high transparency have been reported in the literature, while silica particles can improve performance while preserving optical clarity when dispersion and refractive matching are appropriate [14].

The balance between naturally hydrophilic nanoparticles and hydrophobic low-surface-energy components, the dispersion quality of the nanoparticles, and the coating system’s formulation parameters are some of the interrelated factors that significantly affect the long-term stability and durability of nanoparticle-based wood coatings [20,21]. The development of scalable, ecologically safe coating solutions that provide mechanical reinforcement, chemical resistance, and water repellency at the same time is still a challenge for the wood protection sector, despite tremendous advancements [22]. A potential solution for these restrictions is the use of nanoparticles [23,24]. SiO_2_ nanoparticles can increase surface roughness while maintaining optical qualities (absorbance, reflectance, transmittance, color) because of their chemical stability, transparency, and compatibility with polymer matrices [25,26]. Such nanoparticles can highly enhance weathering performance and resistance to moisture when added to acrylic polymers, without sacrificing aesthetic appeal [27,28]. Because of their transparency, aging stability, and reversibility, acrylic resins like Paraloid B72 are particularly appealing in this context [29,30]. They have already been utilized so far in wood finishing and conservation processes [31,32], though the impact of the combination of SiO_2_ nanoparticles and Paraloid B72 has not been proposed or investigated so far, according to the literature review [33].

Tree-of-heaven (*Ailanthus altissima* Mill.) is a widespread invasive tree species in Europe, which has been described as ecologically important in the European context because of its biomass availability and its unique properties [34]. It is extremely fast-growing, with low requirements in nutrients, climatic conditions and moisture, an underutilized hardwood species that generates woody biomass of low value that requires protection and further improvement. In the current study, for the first time according to the literature, the protection and preservation of this wood species by applying a short-term immersion in Paraloid B72-based syntheses containing different amounts of nano-silica has been attempted. There is very limited published information and published data regarding the tree-of-heaven wood modification potential, and also, the combination of SiO_2_ nanoparticles with Paraloid B72 modification on this or other hardwood species. Even though this wood species is considered of low density and value, it provides an extremely satisfying strength-to-weight ratio [34], and its enhancement through advanced surface treatments could transform this undesirable biomass into value-added structural materials and constructions (furniture, frames, etc.).

More specifically, in this study, Paraloid B72 was dissolved and applied pure or together with SiO_2_ at different shares (introduced at 1–4%), to examine the potential improvement of wood hydrophobicity and resistance to weathering phenomena. FTIR analysis was additionally employed to investigate the chemical characteristics of the formed nanocomposite coatings and the interaction between the acrylic polymer and silica nanoparticles. Treated and untreated specimens were characterized in terms of physical properties (density, equilibrium moisture content, color, surface roughness), hygroscopic properties (swelling and absorption percentage, contact angle) and resistance to accelerated weathering under xenon light exposure with cyclic wetting–drying conditions to assess their performance. This research aims to explore the effectiveness of nanoparticle-enhanced acrylic coatings for the protection of both historical and modern hardwood-based structures.

## 2. Materials and Methods

For this experimental investigation, tree-of-heaven (*Ailanthus altissima* Mill.) was chosen as the substrate because of its widespread availability, increased presence in urban and natural settings, and growing interest in its possible use in structural applications and wood products. Even though this species is regarded as a non-native and rapidly growing species in the European area, its wood has received interest because of its moderate density, workability, and the need to investigate value-added applications, especially in those that require surface protection and durability enhancement.

Two trunks of this species were harvested from the AUTh campus (School of Forestry and Natural Environment) in Thessaloniki (Northern Greece). The trees’ age ranged from 29 to 38 and were selected for their straightness and soundness. The trunk pieces were transported to the laboratory and sawn into boards. Only boards free of apparent defects participated in the experimental work.

**Figure 1:**
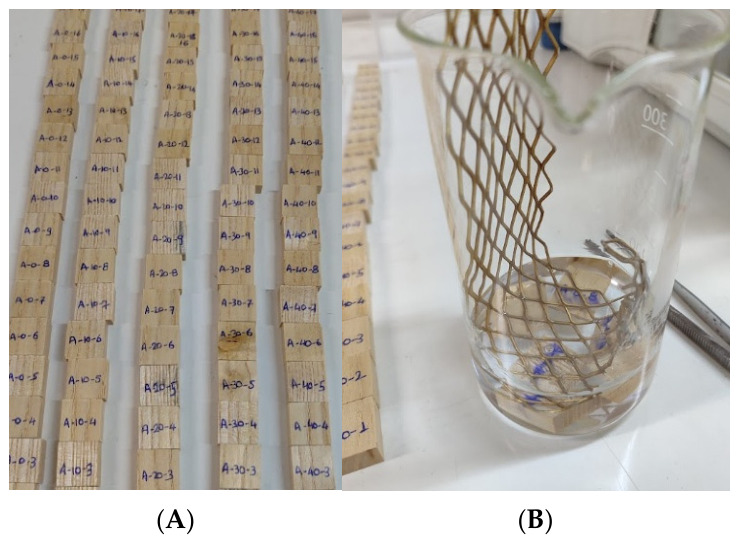
Tree-of-heaven specimens (**A**) and their immersion in Paraloid B72 and nanoparticles solution (**B**).

The boards were conditioned (20 ± 2 °C, 65 ± 1% relative humidity) till constant weight, which corresponded to a moisture content of 8.7–9.8%. The material was machined into specimens with nominal dimensions of 20 × 20 × 10 mm (Figure 1), with the tangential surface representing the largest surface area. 12–15 samples were prepared for each material category. An analytical scale of high accuracy (4 decimals) and a digital caliper (Mitutoyo 500-196-30, Mitutoyo, Kawasaki, Japan) with a resolution of 0.01 mm were used to accurately measure the specimen mass and dimensions of the specimens. All specimens were oven-dried at 75 °C for over 24 h before treatment to calculate their oven-dry mass. They were then kept in sealed bags to maintain their dry state until immersion.

### Application of Surface Treatments

Acetone (C_3_H_3_O, ≥99.5%, Honeywell, Sigma-Aldrich, St. Louis, MO, USA), a solvent with quick evaporation and low toxicity, was used to dissolve Paraloid B72 (ethyl methacrylate–co–methyl acrylate), which was originally supplied in pellet form. A 20:80 concentration ratio (Paraloid: acetone) was used since it is characterized by low viscosity and efficient penetration into the cell walls of the wood, allowing the polymer to be distributed uniformly throughout the wood structure. Paraloid B72 is appropriate for wood surface protection applications because of its solubility properties.

Low amounts of silicon dioxide nanoparticles (SiO_2_; particle size 5–20 nm, nanopowder, 99.5% trace metals, Sigma-Aldrich) were added to motivate penetration into the near-surface wood layers without significantly raising material costs. Keeping the nanoparticle content low was crucial to maintaining the suggested treatment systems’ economic viability.

In order to minimize treatment duration and weight percent gain (WPG), each sample was totally submerged in the Paraloid–nanoparticle solution for a short duration of one minute. The specimens were weighed as soon as they were removed from the synthesized solutions, conditioned under stable conditions and then oven-dried between 24 and 48 h at 80 °C till constant dry-weight, to calculate WPG and solution uptake, using the following equation:

$$\mathrm{WPG}\;(\%)=\left[\frac{m_{of}-m_{oi}}{m_{oi}}\right]\times 100$$

where *m_of_* is the final oven-dry mass of the treated specimen and *m_oi_* is the initial oven-dry mass prior to treatment.

Fourier-transform infrared spectroscopy (FTIR) analysis was performed to investigate the chemical characteristics of untreated and treated wood specimens and to evaluate the incorporation of Paraloid B-72 and SiO_2_ nanoparticles within the wood structure. Spectra were recorded using an FTIR (PerkinElmer Spectrum Two, PerkinElmer, Shelton, CT, USA) spectrometer equipped with an attenuated total reflectance (ATR) accessory in the range of 4000–400 cm^−1^, at a resolution of 4 cm^−1^ and averaging 32 scans per sample. All spectra were collected under ambient laboratory conditions and baseline corrected prior to analysis.

A Minolta colorimeter was used to determine the surface color in compliance with ASTM D1536-58 T1964 [35]. Color was assessed in the CIE Lab* color space, which allows color changes to be quantitatively compared. Lightness is represented by the L* coordinate, which ranges from 0 (black) to 100 (pure white). Chromatic variation along the red–green and yellow–blue axes is described by the a* and b* coordinates, respectively. Each specimen’s color coordinates (L*, a*, and b*) were noted both prior to and after treatment. Color coordinates change usually tracks photochemical modification of lignin-derived chromophores induced by the treatment or weathering process [13,14].

The analysis of possible treatment-induced changes of the wood surface also included the surface roughness evaluation. Surface roughness reflects topographical changes associated with erosion and fiber exposure of the material [14]. All measurements were carried out perpendicularly to the grain within a specified 10 mm-radius area at the center of each specimen surface, comprising many growth rings, to guarantee consistency. Measurements of roughness, including untreated reference samples, were performed both before and after treatment. The measurements were carried out in accordance with ISO 21920-2 [36] and the profilometer manufacturer’s instructions, which described an established procedure that was quite well-documented in the literature. A fine stylus-equipped Mitutoyo Surftest SJ-301 contact profilometer (Mitutoyo Corporation, Kawasaki, Japan) was utilized. The stylus operated at a maximum angle of 90° and traversed at a speed of 10 mm/min. Its tip diameter was 4 μm. Measurements were made throughout a 4.5 mm sampling length. For each specimen, at least 5 measurements were taken, while at least 30 measurements were taken per material category. Therefore, three roughness parameters were recorded: the maximum roughness (*Rq*), the mean peak-to-valley height (*Rz*), and the arithmetic mean roughness (*Ra*). The device was calibrated at room temperature (20 ± 2 °C) using the manufacturer’s reference plate prior to the measurements.

Measurements of static sessile-drop contact angle were implemented on the surfaces of the untreated and treated specimens, since the contact angle reflects surface wettability and hygroscopicity. Static water contact angle measurements were performed using a KRÜSS Drop Shape Analyzer equipped with a CCD camera (KRÜSS GmbH/KRÜSS Scientific Instruments, Hamburg, Germany) and automated dosing system. Sessile droplets of deionized water (3–5 μL) were deposited onto the wood surface using a microsyringe. Images were acquired immediately (within 5 s) after droplet deposition and analyzed using Young–Laplace fitting. Measurements were conducted at room temperature (23 ± 2 °C). At least five droplets were measured per specimen (15 values/material category), and the results are reported as mean and standard deviation values. The droplet volume was 2 μL, and the acquisition time was within 3 s. The artificial weathering of the specimens was also evaluated based on the following process. This test combined cyclic changes in temperature and relative humidity with xenon light irradiation to expedite artificial weathering of both treated and untreated tree-of-heaven specimens in a controlled laboratory setting. In accordance with ISO 4892-2 [37], a weathering exposure test was carried out in a commercial xenon test chamber (SUNTEST XLS+, Atlas Material Testing Technology LLC, Mount Prospect, IL, USA). A xenon light source with an intensity of 200 W/m^2^ and a wavelength range of 290–400 nm was used to irradiate the samples. A black panel at 65 °C and 50% relative humidity in the chamber was employed for the test process conditions. The specimens were placed about 500 mm away from the xenon lamp, and the total exposure time was 4 weeks. After 3 weeks, the test was briefly paused to enable the intermediate characterization of specimens. All samples were assessed for changes in mass, surface color, surface roughness, and longitudinal, radial, and tangential dimensions, following three and four weeks of artificial weathering. The test procedures that were described in the previous paragraphs were applied throughout these measurements after the weathering exposure tests.

One-way ANOVA was performed using SPSS software (version 28, supplied by AUTH University), while results processing and the creation of the experimental results’ diagrams and tables were accomplished using Microsoft Excel. In particular, the Tukey post hoc test of One-Way ANOVA was employed, which concurrently performs the Tukey HDS (Honesty Significance Difference) test to determine precisely which group means vary substantially while accounting for Type I errors (false positives) in all pairwise comparisons. At *p* < 0.05, differences were considered statistically significant.

## 3. Results

### 3.1. Dimensional Changes Induced by the Treatment

**Figure 2:**
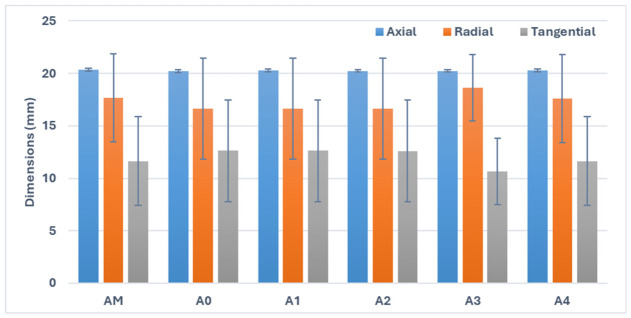
Mean axial, radial, and tangential dimensions of tree-of-heaven (A) for the unmodified reference (AM) and the modified samples (A0: 0% nanoparticles in Paraloid B72, A1: 1% nanoparticles, A2: 2% nanoparticles, A3: 3% nanoparticles, A4: 4% nanoparticles).

After the performance of various impregnation treatments (with Paraloid and combinations of Paraloid with silica dioxide nanoparticles), the samples’ dimensions (length, width, and thickness) remained insignificantly changed from their initial state prior to treatment (Figure 2). There were no noticeable or statistically significant changes in dimensions, brought about by the samples’ brief treatments. This is particularly essential for maintaining the wood quality (avoiding swelling and the usually subsequent cracks, deformations, etc.), applying the treated wood in various high-quality structures, and utilizing these preservatives in historically significant wooden structures that still survive.

Anisotropic behavior, which is typical of wood materials, was evident in all samples. Throughout each modification, the axial dimension remained rather stable, with values about 20 mm and relatively low variance. This demonstrates that, in comparison to transverse directions, axial shrinking or swelling is barely noticeable. The radial and tangential dimensions, on the other hand, displayed considerably lower values and higher fluctuation, indicating that wood anatomy has a dominant influence on transverse dimensional behavior. Length did not significantly differ between AM and the modified samples, suggesting that the longitudinal structure of tree-of-heaven was not significantly affected by the applied changes. Regardless of treatment, the axial direction’s dimensional stability is further supported by the relatively low standard deviations. The range of radial dimension of the samples (width) was measured to be 16–18 mm. In comparison to the reference, modified samples A3 and A4 showed a marginally higher increase in radial dimension (width), indicating a mild increase in radial swelling. The statistical analysis, however, suggests that these variations were not significant and could instead be explained by natural variability as opposed to a significant modification effect. In general, there were low radial dimension changes, indicating a steady reaction to these surface modifications. Out of the three directions, values of tangential dimensions (thickness) swelling were the lowest, usually between 10 and 13 mm, but they revealed the highest standard deviation. The tangential direction of samples, well-known to be slightly more susceptible to dimensional changes [38], is also reflected in this behavior. In comparison to samples A0–A2, samples A3 and A4 had slightly lower tangential dimensions (sample thickness), indicating better dimensional stability in this challenging anatomical direction of wood. This indicates that higher nanoparticle concentrations improved transverse dimensional stability. The findings demonstrate that while modification affects transverse dimensional performance, it does not alter this species’ anisotropic character essentially. Higher levels of nanoparticles appeared to enhance wood dimensional stability (in a brief impregnation applied), which is essential for uses requiring shape-stability in the conditions of the exterior environment (complicated structures, furniture, frames, etc.).

### 3.2. Chemical Characterization

**Figure 3:**
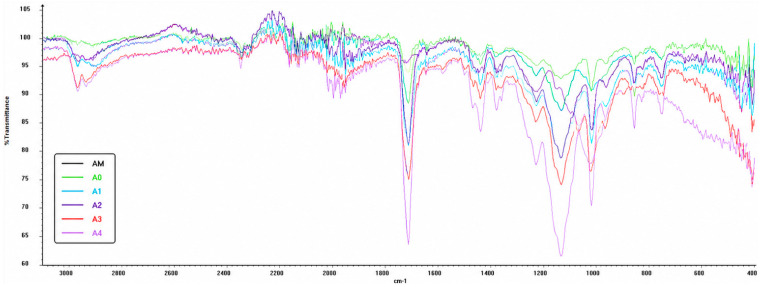
FTIR spectra of untreated (AM) and treated tree-of-heaven wood specimens (A0–A4) modified with Paraloid B72 and different concentrations of SiO_2_ nanoparticles.

The FTIR spectrum of the untreated wood (AM) exhibited the characteristic absorption bands of lignocellulosic materials (Figure 3), including a broad O–H stretching band around 3300 cm^−1^, aliphatic C–H stretching vibrations near 2900 cm^−1^, and C–O–C stretching vibrations at approximately 1030 cm^−1^, corresponding to cellulose and lignin constituents.

Following impregnation with neat Paraloid B-72 (A0), a distinct absorption band emerged at approximately 1730 cm^−1^, attributed to the C=O stretching vibration of the ester groups of the acrylic copolymer, confirming successful deposition of the polymer within the wood structure. A slight reduction and broadening of the O–H stretching band was also observed after treatment, suggesting partial masking of hydrophilic hydroxyl groups by the polymeric coating, which is consistent with the enhanced hydrophobic behavior of the treated specimens.

The incorporation of SiO_2_ nanoparticles (A1–A4) resulted in a progressive increase in the intensity of the absorption bands within the 1000–1200 cm^−1^ region, assigned to asymmetric Si–O–Si stretching vibrations, together with the band near 800 cm^−1^ associated with symmetric Si–O stretching. The enhancement of these bands followed a concentration-dependent trend, with the A4 sample exhibiting the highest absorbance intensity, indicating effective incorporation and good dispersion of silica nanoparticles within the Paraloid matrix. Moreover, the increasing definition of the fingerprint region (1200–900 cm^−1^) suggests the formation of a more homogeneous inorganic–organic nanocomposite coating.

At higher silica concentrations, particularly for A4, the increased intensity of silica-related bands may also indicate localized nanoparticle clustering within the polymer matrix, which could contribute to the enhanced surface roughness and hydrophobic performance observed in the treated samples. Similar behavior has been reported for Paraloid–silica nanocomposite coatings, where silica nanoparticles improve surface stability and protective performance through the development of hierarchical micro/nanostructures.

In general, no significant disappearance or shift of the characteristic wood absorption bands was observed after treatment, indicating that the impregnation process preserved the chemical integrity of the lignocellulosic substrate. Furthermore, the absence of new absorption bands suggests that the interaction between Paraloid B-72 and SiO_2_ nanoparticles is predominantly physical rather than chemical, with no evidence of covalent bond formation or modification of the polymer backbone.

The preservation of the characteristic wood absorption bands after treatment further supports the suitability of the proposed system for conservation-oriented applications, where maintaining the chemical integrity of the original substrate is essential.

### 3.3. Color Changes

**Figure 4:**
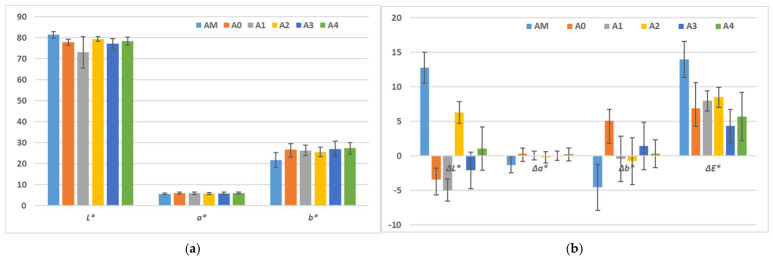
Color coordinates (L*, a*, b*) (**a**) and determined color differences (ΔL*, Δa*, Δb*, ΔE*) of tree-of-heaven wood samples (**b**) for the different treatments (AM, A0–A4).

Figure 4 displays tree-of-heaven color coordinates (L*, a*, b*) and the accompanying differences (ΔL*, Δa*, Δb*, and ΔE*), as a result of treatment. A lighter surface appearance was indicated by the untreated reference sample (AM), which had the highest L* value. The L* values of all modified samples (A0–A4) were marginally lower, indicating a mild darkening impact brought on by the modification procedure. In contrast to A0, A1, A2, A3 and A4 maintained higher L* values among the modified samples, suggesting superior surface lightness preservation/color stability. Chemical and structural alterations in the wood surface improve light absorption, especially after exposure to artificial weathering, and are responsible for the decrease in L*, apart from the potential movement of the extractives in the surface during the immersion [39].

For each of the samples, the a* values remained low, suggesting slight changes along the red-green axis. Some modified samples showed slight increases in a*, indicating limited reddening, though these changes were not relatively noticeable. Coordinate b*, however, displayed more pronounced changes. An increase in yellow hue was indicated by the modified samples’ higher b* values when compared to the reference. This effect is frequently connected to the development of chromophore structures brought about by lignin oxidation and degradation processes during weathering and modification [40].

### 3.4. Surface Roughness

According to the results, surface roughness values were significantly higher in the vertical direction to the grain than in the parallel-to-grain direction for all samples. This was expected due to the transition zones from earlywood to latewood of the growth rings that are encountered by the stylus during the measurement. Wood intrinsic anisotropy and the tangential surface’s increased susceptibility to fiber-lifting, cell wall separation, and microcrack formation during weathering are also reflected in this tendency/finding. Increased surface peak-to-valley heights in the tangential direction were indicated by the *Rz* parameter, which revealed the highest difference between axial and tangential surface roughness. The unmodified sample (AM) showed the lowest roughness values in the parallel to grain direction for all parameters (*Ra*, *Rq*, and *Rz*), suggesting a uniform and smooth surface both before and after exposure. The parallel to grain roughness of the modified samples was somewhat elevated; A3 and A4 had marginally higher *Rz* values, indicating minimal surface roughness increase. All treatments, however, maintained a low total parallel-to-grain roughness, suggesting these surfaces are less susceptible to alteration and artificial weathering.

**Figure 5:**
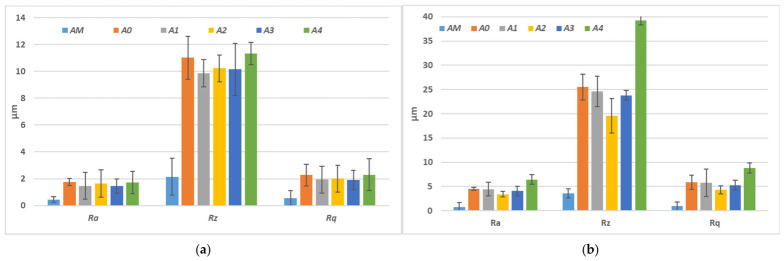
Mean values of surface roughness parameters (*Ra*, *Rq*, and *Rz* in μm) of tree-of-heaven measured in Parallel to grain (**a**) and vertically to grain direction (**b**) for the unmodified (AM) and modified samples (A0–A4).

Tangential surface roughness values, on the other hand, rose for all modified samples, especially the parameter of *Rz* (Figure 5). The samples with the highest tangential *Rz* values, A3 and A4, exhibited intense surface features that cause roughness and more noticeable surface imperfections. Increased photodegradation and moisture-induced stress levels operating perpendicular to the grain are responsible for this increase, as they encourage surface erosion and fiber detachment [41].

Reduced standard deviation in relation to the magnitude of the roughness parameters revealed that modified samples exhibited more consistent surface quality than the reference, despite the higher absolute values. This implies that modification changes the degradation pattern and results in more consistent surface change and surface quality preservation.

Higher modification levels (A3 and A4) caused more noticeable surface roughness increase, particularly in the tangential direction, whereas lower modification levels (A0–A2) produced moderate increases in roughness. This pattern suggests that higher modification intensity (nanoparticles presence) affects surface morphology because of altered cell wall chemistry and decreased wood structural flexibility. However, while having higher roughness values, samples A3 and A4 also revealed higher color and dimensional stability, indicating that controlled surface restructuring rather than general material degradation may be the cause of increased roughness. The findings demonstrate that the degree of alteration and grain orientation have a significant impact on the surface roughness of tree-of-heaven wood. Tangential surfaces were strongly affected by weathering and alteration, but roughness in the parallel-to-grain direction stayed more stable. Surface roughness is not by itself a reliable indication of material durability, as evidenced by the significant coexistence of higher roughness levels in modified samples with enhanced color stability and dimensional stability performance. A thorough evaluation of several properties and characteristics, such as weathering resistance, surface roughness, physical, chemical, optical, hygroscopic properties and dimensional stability characteristics, is necessary to be implemented in parallel.

Assessing potential correlations between roughness and color change of the specimens, one could observe that color stability was both directly and indirectly correlated to surface roughness. Roughness increase that was accompanied by higher hydrophobicity revealed to have a less detrimental effect on color stability, although increased roughness can deteriorate perceptual color shifts and increase light scattering. Because of their decreased surface wettability, sample categories like A3 and A4 maintained low ΔE* values, despite demonstrating increased surface roughness. Accordingly, color stability in tree-of-heaven seems to be controlled by the equilibrium between surface chemistry and roughness in addition to surface topography (sapwood, heartwood, annual ring, earlywood-latewood, etc.).

### 3.5. Contact Angle

**Figure 6:**
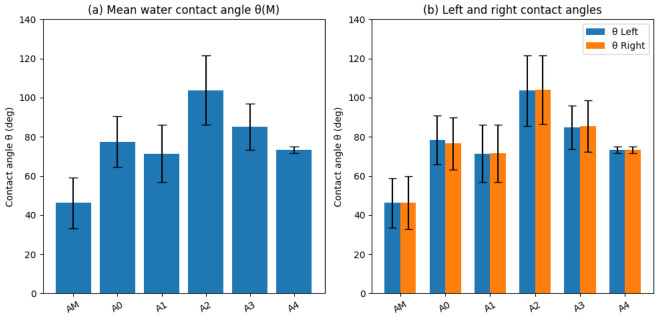
Water contact angle (θ, mean ± SD) of tree-of-heaven specimens for the control (AM) and the treated samples (A0, A1, A2, A3, A4).

**Figure 7:**
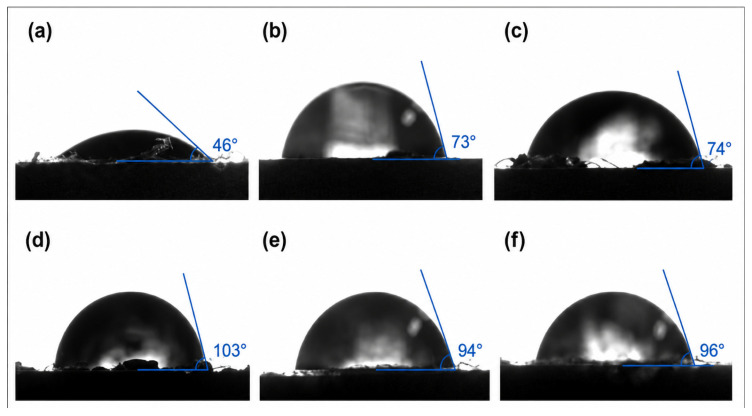
Contact angle images of *Ailanthus altissima* wood specimens: (**a**) untreated, (**b**) 0% SiO_2_, (**c**) 1% SiO_2_, (**d**) 2% SiO_2_, (e) 3% SiO_2_, and (**f**) 4% SiO_2_.

According to contact angle test findings, the unmodified samples (AM) revealed the lowest mean contact angle (θ = 46°), as shown in Figure 6 and Figure 7, suggesting a quite hydrophilic surface. Since exposed hydroxyl groups in cellulose and hemicelluloses encourage robust contact with water, this behavior is typical of untreated wood surfaces [13]. A noticeable decrease in surface wettability, as a result of treatment, was evident in all treated samples (Paraloid and Paraloid-nanoparticles combinations), which displayed a marked rise in contact angle. With the highest mean contact angle (θ = 104°) among them, sample A2 showed a shift toward hydrophobic surface behavior. Samples A3 and A0 also demonstrated increased contact angles (≈85° and 78°, respectively), while A1 and A4 presented moderate values.

For several treatments (particularly A2 and A1), the contact angle measurements’ standard deviations were high, which was indicative of surface heterogeneity as well as regional differences in surface roughness and chemistry. A4 category samples, on the other hand, displayed a low standard deviation, indicating a more consistent and stable surface energy condition. For each sample, the left and right contact angles (θL and θR) showed good agreement, suggesting symmetrical droplet behavior and trustworthy measurement settings. The microstructural characteristics of wood and surface roughness anisotropy may be the cause of minor variations observed in some situations. The reduction of accessible hydroxyl groups, surface densification, and the deposition of less polar chemicals are among the chemical and physical changes at the wood surface that can be responsible for the rise in contact angles after treatment [42]. These modifications increase hydrophobicity by decreasing the surface’s attraction for water. An ideal treatment intensity that maximizes water repellency is suggested by the exceptionally high contact angle seen for A2. Nevertheless, successive treatment level increases (A3 and A4) did not lead to further increases in contact angle, suggesting a potential saturation effect or competing mechanisms such as the growth of surface roughness. These results show that tree-of-heaven surface characteristics can be successfully modified by controlled treatment for uses where moisture resistance is a crucial demand. High standard deviation values in some contact-angle datasets are attributed to surface heterogeneity and local differences in roughness and chemistry. In general, contact-angle variability on wood is considered to be strongly influenced by roughness, anisotropy, and chemical heterogeneity [43]. Additionally, the FTIR findings, combined with the increased contact-angle values and the evolution of surface roughness, suggest that the Paraloid–silica system formed a hierarchical protective coating capable of reducing surface wettability while improving resistance to moisture-induced degradation.

The findings demonstrate a correlation between wettability, surface roughness, and color stability. Increased contact angle treatments limited photodegradation and color change by reducing water–surface interactions. Although treatment increased surface roughness, improved hydrophobicity counteracted its negative impact on color stability. The most advantageous balance between high contact angles, regulated roughness, and decreased ΔE* was attained by intermediate to advanced modification levels among the treatments under study. These results emphasize how crucial it is to consider both surface chemistry and surface morphology when developing treatments to enhance the long-term performance of tree-of-heaven or of other wood species. Although higher nanoparticle concentrations increased surface roughness, this effect was accompanied by improved hydrophobicity and enhanced resistance to photodegradation, indicating a beneficial balance between surface texturing and protective performance.

### 3.6. Artificial Weathering

**Figure 8:**
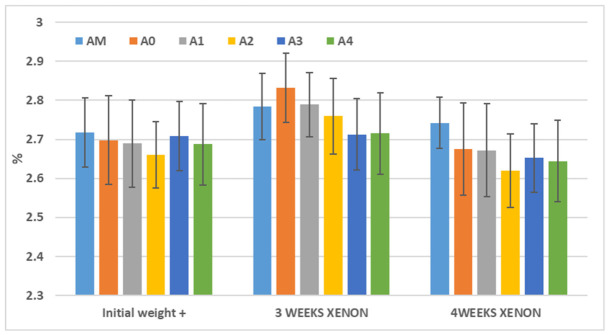
Mass changes among the different categories due to treatments (AM: untreated; A0: Paraloid only; A1: impregnated with 1% SiO_2_; A2: impregnated with 2% SiO_2_; A3: impregnated with 3% SiO_2_; A4: impregnated with 4% SiO_2_ and exposure time under xenon artificial weathering (initial state, 3 weeks, and 4 weeks).

Prior to treatment and weathering, all specimens showed similar initial weights (≈2.65–2.72 g), indicating good sample consistency. This demonstrates that treatment and exposure, not sample variability, are the cause of subsequent mass differences. Regarding the impact of xenon exposure, all modified samples demonstrated a noticeable rise in mass over their starting points after three weeks (Figure 8). The main cause of this mass gain is moisture uptake brought on by advanced surface oxidation and UV-induced hydrophilic functional group synthesis on lignin and hemicelluloses. The highest water uptake among the treatments was observed in A0 and A1, indicating that these alterations resulted in surfaces with a higher affinity for water following first weathering (3-week duration), though without marking statistically significant differences.

Following the 4-week exposure, all treatments’ samples showed a general slight decrease in mass, as compared to the 3-week corresponding values. The masses, however, continued to be somewhat higher than or right around the starting point levels. The partial leaching of low-molecular-weight breakdown products, washout of extractives, surface erosion, and microcracking, which prevent additional moisture retention, can all be used as potential reasons to explain this mass reduction. From week 3 to week 4, samples of the A2 and A3 categories displayed the highest decline (although the difference was not statistically significant), suggesting a decreased ability to withstand prolonged photodegradation. A4 samples’ steady mass, on the other hand, suggests that it is more resistant to material loss brought on by weathering. A balance between material loss and moisture sorption during weathering is reflected in the mass change. In line with their enhanced dimensional stability and surface roughness control noted in previous measurements, treatments that restricted water intake and slowed surface deterioration (such as A3 and A4) showed more stable mass values.

**Figure 9:**
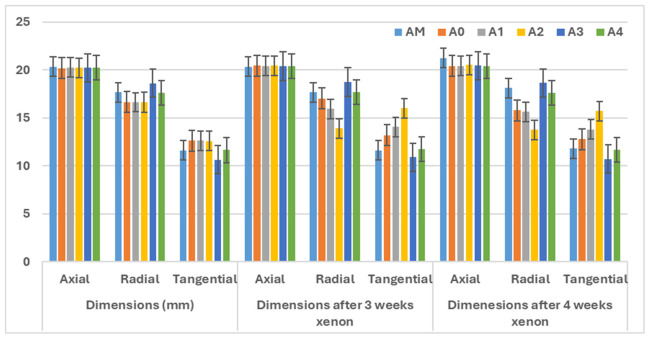
Change of the axial, radial, and tangential dimensions (mm) of tree-of-heaven specimens before exposure, after 3 weeks, and after 4 weeks of xenon artificial weathering for all treatments (AM, A0–A4).

All treatments displayed relatively similar dimensions in all three anatomical directions prior to exposure, as seen in Figure 9. All specimens showed dimensional changes following three weeks of Xenon exposure, with the biggest modifications taking place in the tangential and radial directions and the least amount in the axial direction. Wood exhibits this behavior because transverse orientations are far more susceptible to photodegradation and moisture absorption. Higher levels of moisture absorption and swelling were indicated by the control (AM), which displayed the highest rise in both radial and tangential dimensions. The swelling and absorption percentage values provide direct information about the dimensional stability and moisture sensitivity of the materials [1]. The treated samples, especially of categories A2, A3, and A4, on the other hand, had fewer dimensional changes, indicating that the treatments were successful in reducing hygroscopic swelling and dimensional deformation. In comparison to the 3-week values, most treatments showed a partial reduction in dimensions after 4 weeks of exposure. This decrease is explained by partial drying following photodegradation, leaching of low-molecular-weight chemicals, and loss of degraded surface material. The control samples’ lower dimensional stability was confirmed by the fact that, despite this decline, they still showed higher dimensional variability than the treated ones. After extended exposure, the samples of treatment categories A2, A4 and to a lesser extent A3 exhibited the most consistent behavior over time, keeping their dimensions constant (the most stable contact-angle or ΔE* behavior, with trade-offs between hydrophobicity, color stability, roughness evolution, with a possible embrittlement at higher nanoparticle loading). While axial dimensions (length) stayed unchanged, the tangential direction (thickness) showed the highest relative changes, followed by the radial direction (width). This demonstrates that the transverse wood structure, which is more vulnerable to moisture and photochemical deterioration, was the most affected by xenon weathering.

The initial lightness was highest in the untreated samples (AM), suggesting brighter surfaces. All treated samples, with or without nanoparticles (A0–A4), displayed a decrease in L* upon exposure, which is indicative of surface darkening brought on by lignin photodegradation and the creation of chromophore chemicals. A1 and A3 showed the highest reduction in L* among the treated samples, whereas A2 and A4 maintained higher lightness values, indicating superior resistance to UV-induced darkening. Both a* and b* values varied moderately across all treatments. Due to lignin oxidation, UV-exposed wood typically exhibits yellowing, as indicated by an increase in b*. While A3 and A4 showed more modest fluctuations, indicating higher color stability, A0 and A1 showed the highest increase in b*. The a* coordinate remained constant, suggesting that the yellowing impact outweighed any red-green shifts.

**Figure 10:**
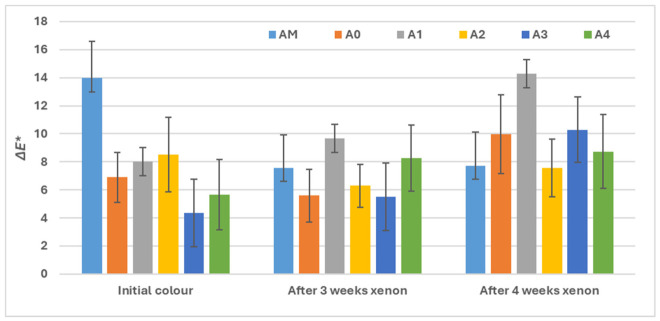
Total color difference (ΔE*) of the untreated specimens (AM) and treated ones (A0–A4) before exposure, after 3 weeks, and after 4 weeks of exposure to xenon artificial weathering.

The performance of the treatments is readily distinguished by the total color difference ΔE* (Figure 10). Strong visual degradation was evident in the reference (AM) specimens, which had the highest ΔE*. Color changes in weathered wood are usually closely related to lignin degradation, chromophore formation, bleaching, and subsequent leaching phenomena [44,45]. A0 and A1 revealed comparatively high ΔE* values among the treated samples, whereas A3 and A4 had the lowest values, indicating superior color stability during xenon exposure, and A2 exhibited intermediate behavior, indicating partial but subpar protection against photodegradation. All samples recorded an increase in ΔE* after 3 weeks, indicating that photodegradation had begun. The highest ΔE* values were measured in the control and A1 samples, suggesting that lignin breakdown and the production of colored degradation products caused rapid discoloration.

A3 and A2, on the other hand, recorded the lowest ΔE* values, indicating higher resistance to color change brought on by UV light. All treatments led to a further increase in ΔE* after 4 weeks, although the degree of change varied significantly amongst them. Severe discoloration was indicated by the highest ΔE* values in control (AM) and A1. While A2 displayed intermediate behavior, A3 and A4 retained much lower ΔE*, indicating improved long-term color stability.

The findings show that tree-of-heaven color stability is markedly enhanced by surface modification. With the lowest ΔE* values, treatments A3 and A4 were the most successful in preventing yellowing and darkening. These results are in line with their increased dimensional stability, decreased moisture uptake, and better surface hydrophobicity.

**Figure 11:**
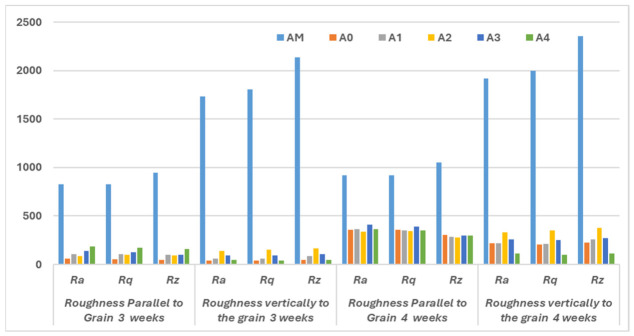
Evolution of the surface roughness parameters *Ra*, *Rq*, and *Rz* (μm) of *Ailanthus altissima* for the untreated control (AM) and the modified samples (A0–A4) before exposure, after 3 weeks, and after 4 weeks of Xenon artificial weathering.

All specimens had low and comparable surface roughness values prior to weathering, with *Ra* and *Rq* usually being below 3 μm and *Rz* below 12 μm. The slight variations between treatments show that the original surface quality was not considerably deteriorated by the treatment. All samples showed a noticeable rise in all roughness indicators after the 3 weeks of exposure to weathering (Figure 11). The peak-to-valley parameter *Rz* showed a particularly noticeable rise, suggesting the development of deeper surface imperfections brought on by photodegradation, microcracking, and early-stage erosion. Due to surface smoothing brought on by the removal of weakly bonded material, the untreated control (AM) showed the least increase in roughness. The treated samples, particularly of categories A3 and A4, on the other hand, displayed noticeably higher *Rz* values, suggesting localized deterioration of the modified surface layer and increased surface structure. Roughness intensified further for all treatments after 4 weeks, with *Rz* values increasing significantly, especially for A3 and A4 samples. This is a sign of advanced crack growth and surface erosion. Progressive micro-scale surface deterioration is confirmed by the ongoing rise in *Rq* and *Ra.* A4 treatment samples recorded the highest roughness value among the treatments, indicating that while it offers adequate chemical protection, extended UV exposure makes its surface layer more brittle and prone to cracking. The results demonstrate that tree-of-heaven undergoes progressive surface degradation due to xenon weathering, mostly manifested as an increase in surface roughness. Although surface modification increases color stability and moisture resistance, it also creates a more brittle surface layer that gets rougher with extended UV exposure. The observed changes in wettability and color are closely related to the evolution of roughness: higher roughness encourages water retention and light scattering, which speeds up photodegradation and raises ΔE* values over extended exposure times.

**Figure 12:**
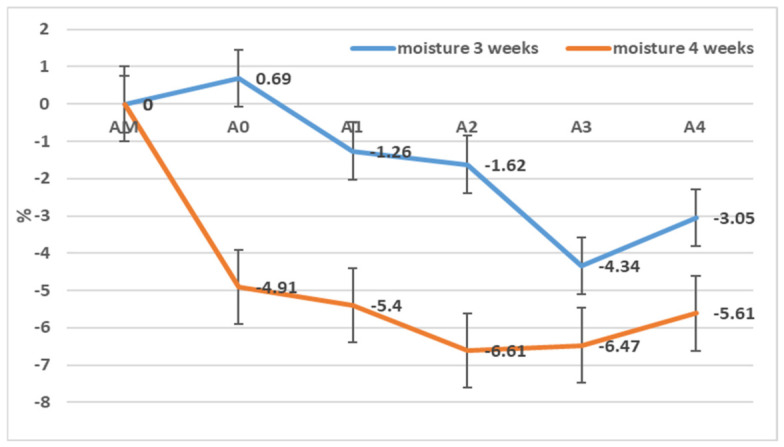
Moisture content change (%) of tree-of-heaven specimens after 3 and 4 weeks of Xenon artificial weathering for the control (AM) and the treated samples (A0–A4).

The untreated samples (AM) displayed very low moisture content levels (near zero) after three weeks, suggesting minimal moisture uptake (Figure 12). Moreover, the treated samples showed a decreasing moisture content as they showed an increasing number of negative moisture readings. This pattern indicates that the wood surface’s hygroscopicity was successfully reduced by the treatments, with higher intensity treatment levels (A2–A4) offering higher resistance to moisture sorption.

All samples demonstrated lower moisture content values after four weeks, suggesting that photodegradation and surface modification had further reduced moisture retention. A4-treated samples displayed somewhat higher moisture content levels, indicating some surface cracking or microstructural deterioration that partially restored water accessibility, but A2 and A3 samples showed a significant reduction in moisture.

All treatments exhibited a gradual decrease in moisture content from 3 to 4 weeks, indicating that extended exposure to xenon causes hygroscopic components to gradually dry up and deteriorate. Even after prolonged UV exposure, the relative ranking of treatments stayed consistent, demonstrating that the degree of modification impacts moisture resistance.

It was revealed that surface treatments substantially reduced tree-of-heaven’s moisture affinity. The best moisture barrier is provided by intermediate to high modification levels (A2–A4), yet prolonged weathering might somewhat counteract this advantage because of surface erosion and cracking.

These moisture patterns align with the higher contact angles recorded for the same samples, as well as the noted improvement in dimensional stability.

**Figure 13:**
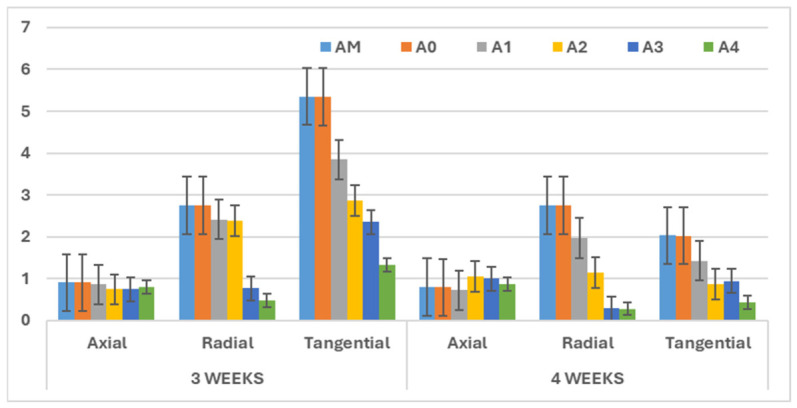
Swelling (%) of tree-of-heaven in axial, radial, and tangential directions after 3 and 4 weeks of xenon artificial weathering for the untreated (AM) and treated samples (A0–A4).

All specimens showed significant anisotropic swelling (Figure 13), with considerably greater dimensional changes in the tangential and radial directions than in the axial direction. With high swelling values in both transverse directions, the untreated samples (AM) displayed the lowest dimensional stability. All treated samples, on the other hand (with Paraloid and Paraloid combined with SiO_2_ nanoparticles), displayed lower swelling, demonstrating how well the modification managed to prevent moisture-induced deformation/swelling, compared to untreated wood. Increasing treatment intensity clearly improved dimensional stability; nevertheless, A0 and A1 only moderately reduced swelling as compared to the control samples. Radial and tangential swelling were significantly reduced in the A2 and A3 sample categories. Even after extended exposure to xenon, A4 samples exhibited the lowest swelling values in all directions, marking quite low dimensional changes. This suggests that moisture penetration and cell-wall swelling were significantly inhibited by the more intense treatment levels. Swelling values were only slightly higher after 4 weeks of xenon exposure than after 3 weeks, especially in the tangential and radial directions. The rise is explained by partial loss of the protective surface layer, microcracking, and increasing surface degradation, all of which promote moisture accessibility. Of course, weathering-induced wetting–drying cycles are known to generate stresses, checks, cracks, and moisture-related deformation, especially in exposed transverse-related directions and porous zones [46]. However, even after 4 weeks of exposure, the heavily treated samples (A3 and A4) continued to exhibit significantly higher dimensional stability than the respective mild-treated and untreated samples. The swelling results show that tree-of-heaven dimensional stability is enhanced by surface modification. These treatments offer the highest defense against moisture- and UV-induced degradation, as evidenced by the significant decrease in transverse swelling for A3 and A4 treatments, which is consistent with their higher contact angles, reduced moisture uptake, and enhanced color stability. Treatments considerably increased color stability at 3 weeks in comparison to the control, with A3 and A4 exhibiting the largest ΔE* reduction (≈69% and ≈59%). At three weeks, there was a negative correlation between ΔE* and tangential roughness (*Ra* and *Rz*) across treatments, suggesting that improved color retention was associated with increased surface roughness. Treatments ranking changed after 4 weeks; A1 showed the highest discoloration, indicating late-stage breakdown of the protective effect, whereas A2 showed the lowest ΔE*.

Respectively, Muhcu et al. [47], who incorporated nano-CuO, nano-ZnO, nano-B_2_O_3_, nano-TiO_2_, and nano-CeO_2_ dispersed in Paraloid B72 (PB72) in Scots pine wood samples, observed that treatments improved water resistance of treated specimens; however, the treatments only delayed water absorption for the first 2 h immersion, and specimens absorbed more water after 24 h immersion. Wood specimens treated with PB72 by immersion absorbed almost the same amount of water as the untreated ones. Of course, the conditions of impregnation and the nanoparticles were different in this study of Muhcu et al. [47]. The authors also referred that overall, for improved water-resistance and biological performance of nanoparticles, PB72 treatments, higher retention levels of nanocompounds and higher WPG values of PB72, along with higher concentration of Paraloid (they had used only 10%) might be effective. Furthermore, Salem et al. [48], who impregnated acacia wood with Paraloid and nanoparticles of TiO_2_ (5% and 10%), observed significant antifungal activity against three studied molds that colonize wood and propose this combination as a preservation method, especially for mold growth prevention on wood surfaces. Finally, Mantanis et al. [49] evaluated the resistance of black pine wood vacuum-treated with zinc oxide, zinc borate and copper oxide nanoparticles against mold and decay fungi and the subterranean termites and concluded that nanozinc borate-treated samples possessed favorable properties, that is, inhibition of termite feeding and decay by *T. versicolor* and presented much higher efficacy than copper-based treatments.

## 4. Conclusions

Surface modification with Paraloid B72 and SiO_2_ nanoparticles significantly improved the hydrophobicity, dimensional stability, and weathering resistance of tree-of-heaven wood. Water contact angle increased from approximately 46° in untreated specimens to values as high as 104° after treatment, demonstrating a significant enhancement in surface hydrophobicity. Treatments containing 2–4% SiO_2_ nanoparticles (A2–A4) exhibited the best overall performance, showing reduced moisture uptake, lower swelling values, and improved color stability during xenon weathering, with A3 and A4 presenting the lowest ΔE* values after accelerated aging. Although surface roughness increased after prolonged exposure, treated specimens maintained superior resistance to photodegradation and dimensional changes compared with untreated wood. FTIR characterization demonstrated the effective formation of Paraloid–silica nanocomposite coatings within the wood structure, contributing to enhanced hydrophobicity and surface protection without altering the chemical integrity of the substrate. In general, silica–acrylic nanocomposite coatings appear to be a promising strategy for improving the durability and weathering resistance of low-value hardwood species, while also offering potential applications in sustainable wood protection and cultural heritage conservation.

## Data Availability

Available upon request to the corresponding author.
